# Mizan sleep quality and Sleep Hygiene Index MiSQuaSHI: a psychometric investigation

**DOI:** 10.5935/1984-0063.20200052

**Published:** 2021

**Authors:** Md Dilshad Manzar

**Affiliations:** Department of Nursing, College of Applied Medical Sciences, Majmaah University, Majmaah, 11952, Saudi Arabia.

**Keywords:** Sleep, Anxiety Disorders, Ethiopia, Sleep Hygiene, Students, Young Adult

## Abstract

**Objective:**

Poor sleep quality and unhealthy sleep hygiene practices are often correlated and co-existing. However, there is no single validated tool to assess both sleep quality and sleep hygiene practices. Therefore, this study presents psychometric validation findings of an instrument developed to assess both sleep quality and sleep hygiene practices.

**Material and Methods:**

A sample (n=395, age = 21.9±4.2 years, body mass index = 20.86±3.22kg/m2, male = 328, female = 67) from Mizan-Tepi University, Ethiopia, completed this cross-sectional study involving the perceived stress scale 10 (PSS -10), the generalized anxiety disorder-7 scale (GAD-7), the Mizan sleep quality and sleep hygiene index (MiSQuaSHI), the Leeds sleep evaluation questionnaire-Mizan (LSEQ-M) and a socio-demographics tool.

**Results:**

No signiﬁcant skewness, kurtosis, and ceiling/ﬂoor effect were seen in the MiSQuaSHI total score. The discriminative validity was favored by significant differences (8 item scores, MiSQuaSHI total and factor scores) in students with moderate-severe GAD than normal. The concurrent validity test found an area under the curve (AUC) of 0.81 (CI 0.76-0.85; p<.0001) with a sensitivity and specificity of 62% and 88%, respectively at the cut-off score of 7.5 with the LSEQ-M. The divergent validity was evidenced by correlations of MiSQuaSHI total score with both GAD -7 (r=0.24) and PSS -10 (r=0.20). The internal consistency was adequate (Cronbach’s alpha=0.70). Exploratory factor analysis (EFA) results were inconsistent. However, confirmatory factor analysis (CFA) found that the 5-factor model had the best ﬁt.

**Discussion:**

The findings support the validity of the MiSQuaSHI as a global measure of poor sleep quality associated with poor sleep hygiene.

## INTRODUCTION

Sleep hygiene is often inherent and an inalienable aspect of sleep quality. Poor sleep quality is associated with poor sleep hygiene^1^^-^^3^. Adolescents and young adults are seemingly at the focal point of this relationship between poor sleep and poor sleep hygiene^1^^-^^4^. Poor sleep hygiene is expressed and exaggerated by the performance of routine life activities in ways that compromise sleep quality and daytime alertness^4^. Such activities are incompatible with the principles of sleep organization and increase arousal at night^4^. Poor sleep hygiene is usually present as an associated condition in insomnia, excessive daytime sleepiness, nocturnal eating syndrome, circadian rhythm sleep disorder, and delayed sleep phase syndrome^4^. Moreover, recent evidence further establishes the relationship between poor sleep hygiene and insomnia in young adults^5^, sleepiness, depression, poor quality of life in obstructive sleep apnea^6^, sleep problems in attention deficit hyperactivity disorder^7^, and short sleep duration in asthmatic patients^8^.

Sleep hygiene interventions may help address the growing public health concern of sleep complaints^9^. Recent pieces of evidence from interventional studies with targeted modification in sleep hygiene practices have shown efficacy in the management of insomnia^10^^-^^12^, insomnia in fibromyalgia^13^, attention deficit hyperactivity disorder^11^, anxiety^12^, and neurodevelopmental disabilities^14^. Enhanced understanding of the impact of sleep hygiene habituation on sleep may help in the development of useful practical guidelines for interventional paradigms^9^.

Previous tools to measure sleep hygiene simply intended to develop a measure of altered behavioral practices. This is evident from the absence of items in those questionnaires, which take account of actual sleep disturbances caused by those factors^15^. It may not be out of place to stress that sleep disturbances that result from altered sleep hygiene practices are the most important clinical features and not just the altered practices themselves^4^. Moreover, poor sleep quality associated with poor sleep hygiene practices usually has associated daytime symptoms, which may compromise social, occupational or cognitive daytime functioning like problems with mood, motivation, attention, vigilance, concentration, and fatigue^4^. Some of the previous sleep hygiene tools have failed to include items to address these conditions^15^.

Questionnaire tools measuring only sleep hygiene practices have suffered from low internal consistency^15^. This may partly be explained by the unrelated nature of the items, which need to be listed together to screen any prospective factor interfering with sleep hygiene^15^. However, non-inclusion of items to address associated changes in sleep quality may also have had a poor bearing on the internal consistency of such tools. To the best of author’s knowledge, there is no rigorously validated single tool to address sleep disturbances as well as altered sleep hygiene practices. Therefore, this study presents psychometric validation findings of a tool developed to assess sleep quality associated with sleep hygiene practices.

## MATERIAL AND METHODS

### Participants and study design

The study sample were selected by a simple random sampling method from Mizan-Tepi University (MTU), Bench Maji Zone, Mizan-Aman, Ethiopia, for a cross-sectional study. The findings from a sample size of 395 students (age = 21.9±4.2 years, body mass index = 20.86±3.22kg/m^2^, male = 328, female = 67) who completed the study are presented in this study. The study was approved by the Institutional Ethical Committee, College of Medicine and Health Sciences, Mizan-Tepi University, Ethiopia. Inclusion criteria was active registration as a regular student of the MTU. Exclusion criteria comprised self-reported memory problems and the use of neuro-psychotic drugs at the time of the study. The investigators explained the purpose and procedure of the study to the participating students. The participants provided informed written consent per the Helsinki declaration.

Amharic is the national language of Ethiopia. Although most students can speak Amharic, the reading proficiency varies. There are about eighty languages and related linguistic ethnicities in the country. English is the medium of instruction in Ethiopian federal universities. Therefore, the English versions of the questionnaires were used in this study. The generalized anxiety disorder-7 scale (GAD-7)^16^, the Leeds sleep evaluation questionnaire-Mizan (LSEQ-M)^17^, the Mizan sleep quality and sleep hygiene index (MiSQuaSHI), the perceived stress scale 10-item scale (PSS-10)^18^ and a socio-demographic questionnaire were administered.

### Measures

#### Generalized Anxiety Disorder-7

The generalized anxiety disorder-7 (GAD-7) is a self-reported tool based on the diagnostic and statistical manual of mental disorders-IV-TR to measure the level of anxiety^16^. All 7-items quantify different symptoms of the respondents’ anxiety and are scored from 0 (not at all) to 4 (nearly every day). The scores of all the seven items are added to generate a total score in the range of 0 to 21. Higher total scores imply an increasing level of anxiety during the last two weeks immediately preceding the test administration^16^.

#### Leeds sleep evaluation questionnaire-Mizan (LSEQ-M)

The Leeds sleep evaluation questionnaire (LSEQ-M) is a self-reported questionnaire to measure sleep quality. There are 10-items, which are 100mm visual analog Likert scales. Zero represents disturbed sleep, and ‘100’ indicates normal sleep. The reported values for all the items are scaled down to 0-10 by dividing the reported scores by 10. These are added to generate the LSEQ-M total score in the range of 0-100. Lower total scores indicate an increasing level of poor sleep^17^.

#### Mizan sleep quality and sleep hygiene index (MiSQuaSHI)

The Mizan sleep quality and sleep hygiene index (MiSQuaSHI) is an 18-item questionnaire developed to assess sleep quality associated with sleep hygiene practices. There are four items to measure sleep disturbances based on the International Classification of Sleep Disorders^4^. Two of these items are dichotomous and scored as yes (1)/no (0). While the two other items are structured categorical variables (item-3 and item-4) scored as 0-3. There are thirteen items to assess sleep hygiene behavior adopted from the sleep hygiene index (SHI). These items measure sleep hygiene behavior according to the International Classification of Sleep Disorders, revised criteria (ICSD-2)^15^. Nine items were incorporated without modifications, while four items (item-5, 7, 9, 12 of the SHI) were added as sub-items of item-14 of the MiSQuaSHI. These four sub-items were initially scored by respondents as dichotomous yes (1)/no (0) measures. However, the respondents’ scores for all four sub-items were added to get a score in the range of 0-4. This was further converted to a dichotomous measure as: ‘0-1’ coded finally as ‘0’, while ‘2-4’ were finally coded as ‘1’. MiSQuaSHI total score is generated by adding the final dichotomous scores for the twelve items i.e., item-1, 2, 5-14 and structured categorical scores for two items i.e., item-3 and 4 (these two items have a score range of 0 to 3) (Supplement 1). Higher MiSQuaSHI scores indicate a higher level of poor sleep quality associated with poor sleep hygiene.

#### Perceived stress scale -10

The perceived stress scale -10 (PSS -10) is a self-reported measure of psychological stress level. There are 10-items scored from 0 (never) to 5 (very often). The scores for all items are pooled to generate a total score in the range of 0 to 40. Higher total scores indicate the increasing level of psychological stress in the respondent in the month preceding the test^18^. PSS -10 has been found to have adequate psychometric validity in the Ethiopian university students^19^.

### Statistical analysis

Most of the analysis was performed by SPSS 23.0 for Windows (SPSS Inc., Chicago, USA). Parallel analysis (Monte Carlo PA) was performed with SPSS 23.0 (SPSS Inc., Chicago, USA) using syntax. Descriptive statistics like frequency, mean with standard deviation, percentage, skewness, and kurtosis index were employed for presenting participant characteristics and distribution assessment of the MiSQuaSHI total score. Cronbach’s alpha test evaluated internal consistency. Spearman’s correlation test was used to measure internal homogeneity (between item score and MiSQuaSHI total score) and divergent validity (between MiSQuaSHI total score/factor scores with GAD -10 and PSS -10). Independent t-test and Mann Whitney U test were used for discriminative validity. The concurrent validity of the MiSQuaSHI was assessed by a receiver operating characteristic (ROC) curve. The MiSQuaSHI total score was the test variable and the LSEQ-M dichotomous variable (i.e., groups with normal sleep and sleep disturbances) was the state variable. The area under the curve (AUC), cut-off score, sensitivity, and specificity were assessed.

Exploratory factor analysis (EFA) for an initial solution was performed for an unrotated solution with principal component analysis extraction. Finally, EFA was performed by principal component analysis extraction with varimax rotation. Cumulative variance rule (>40%), Kaiser’s criteria (Eigenvalue≥1), parallel analysis (Monte Carlo PA) and scree test were used for factor extraction in EFA. The sample adequacy and sample suitability for factor analysis were assessed by the anti-image matrix, Bartlett’s test of sphericity, communality (principal component analysis extraction with unrotated solution), determinant, inter-item correlation and Kaiser-Meyer-Olkin test of sampling adequacy (KMO). Confirmatory factor analysis (CFA) using maximum likelihood extraction and bootstrapping with standardized estimates of factor loading was performed. CFA was attempted on five models, i.e., 1-factor model, 2-factor, 3-factor, 4-factor, and 5-factor. The 2-factor model was assessed based on the theoretical construct of the MiSQuaSHI comprising of two factors namely sleep disturbances and sleep hygiene. While, three models, i.e., 3-factor, 4-factor, and 5-factor were the outcomes of EFA in this study. The 3-factor model did not run with bootstrap to compute standardized regression weights between two variables because variances for one of them failed to be positive. Therefore, the 3-factor model was run without bootstrap. Model fit was assessed by employing multiple indices from different classes of fit measurements^20^. Model fit was assessed by parsimony normed fit index (PNFI), incremental fit index (IFI), comparative fit index (CFI), goodness of fit index (GFI), root mean square residual (RMR), and root mean square error of approximation (RMSEA).

## RESULTS

### Participants’ characteristics

The mean MiSQuaSHI total score was 6.3±3.1 with a range of 0-14. About one-quarter of the students did not have normal BMI, i.e., were under-weight, over-weight or obese. Amhara and Oromo together accounted for about 63% of the participant students. Most of them (72.4%) had either one or two year of university education. More than one-quarter of the participants (28.3%) were from a low or very low-income family (Table 1).

**Table 1:** Participant characteristics.

Characteristics	Mean±SD/ Frequency
**Age**(yr)	21.9±4.2
**BMI**	
Under-weight	65(16.5%)
Normal	296(74.9%)
Over-weight	29(6.3%)
Obese	9(2.3%)
**Ethnicity**	
Bench	32(8.1%)
Kaffa	14(3.5%)
Oromo	117(29.6%)
Amhara	131(33.2%)
Tigre	3(0.8%)
Wolaita	6(1.5%)
Others (including Nuer)	92(23.3%)
**Years of university education**	
One	140(35.4%)
Two	146(37.0%)
Three	43(10.9%)
Four and Above	66(16.7%)
**Gender**	
Male	328(83.0%%)
Female	67(17.0%)
**Monthly Family Income(In Birr)**	
Very Low (less than 445)	44(11.1%)
Low (446-1200)	68(17.2%)
Average (1201-2500)	52(13.2%)
Above average (2501-3500)	28(7.1%)
High (greater than 3500)	77(19.5%)
Undisclosed	126(31.9%)
**MiSQuaSHI total score**	6.3±3.1
**PSS -10**	19.4±6.6
**GAD-7**	7.2±4.4
**LSEQ-M**	58.6±21.2

BMI: Body mass index; MiSQuaSHI: Mizan sleep quality and sleep hygiene index; LSEQ-M: Leeds sleep evaluation questionnaire-Mizan; GAD-7: Generalized anxiety disorder-7 item scale; PSS -10: Perceived stress scale-10-item scale.

### Preliminary item analysis

MiSQuaSHI total score did not show ceiling or floor effect as none and 1.5% of students reported the highest score (i.e., 18) and lowest score (i.e., 0), respectively^21^^,^^22^. There was no significant issue of skewness (skewness index=0.23; standard error of skewness=0.12, z=1.89) or kurtosis (kurtosis index=-0.54; standard error of kurtosis=0.24, z=-2.20) in the distribution of the MiSQuaSHI total score.

### Discriminative validity of the MiSQuaSHI

The discriminative validity test showed that: (i). 8 out of the 14 MiSQuaSHI item scores (1-4, 7, 8, 10, 13); (ii). MiSQuaSHI factor scores, i.e., sleep disturbances and sleep hygiene; and (iii). MiSQuaSHI total score differed significantly between students with moderate-severe GAD than normal (Table 2).

**Table 2:** Internal consistency, homogeneity and communality, and discriminative validity: comparison of the Mizan sleep quality and sleep hygiene index (MiSQuaSHI) scores between normal and those with moderate-severe GAD as determined by GAD-7 scale in Ethiopian university students.

Items of the MiSQuaSHI	Item-total correlatio	Cronbach's Alpha if item deleted	Communality (h2)	Mean rank		*p* value
				Normal (n=118)	Moderate- severe GAD (n=92)	
MiSQuaSHI item-1	.73*	.64	.89	95.58	118.23	<0.01
MiSQuaSHI item-2	.66*	.65	.80	97.85	115.32	<0.01
MiSQuaSHI item-3	.71*	.64	.81	96.90	116.53	<0.01
MiSQuaSHI item-4	.49*	.68	.51	101.18	111.04	.03
MiSQuaSHI item-5	.37*	.70	.49	100.85	111.47	.12
MiSQuaSHI item-6	.29*	.70	.66	103.08	108.61	.40
MiSQuaSHI item-7	.33*	.70	.59	99.28	113.48	.05
MiSQuaSHI item-8	.47*	.68	.47	97.64	115.58	.01
MiSQuaSHI item-9	.31*	.70	.61	105.13	105.98	.87
MiSQuaSHI item-10	.43*	.69	.28	93.25	121.21	<0.01
MiSQuaSHI item-11	.33*	.70	.74	102.08	109.88	.24
MiSQuaSHI item-12	.41*	.69	.69	103.32	108.29	.49
MiSQuaSHI item-13	.32*	.70	.43	90.55	124.67	<0.01
MiSQuaSHI item-14	.28*	.70	.56	100.57	111.83	.10
Sleep disturbances				95.74	118.02	<0.01
Sleep hygiene				89.04	126.61	<0.01
MiSQuaSHI total score$				4.94±2.85	6.96±2.69	<0.01

* *p*<0.01; #Exploratory Factor analysis (EFA) with Principal Component Analysis extraction for unrotated method for initial solution was performed;

$ Mean±SD, Independent t-test was used for the MiSQuaSHI total score and Mann-Whitney U test was applied for component scores; GAD: Generalized anxiety disorder.

### Concurrent validity of the MiSQuaSHI

**Figure 1:**
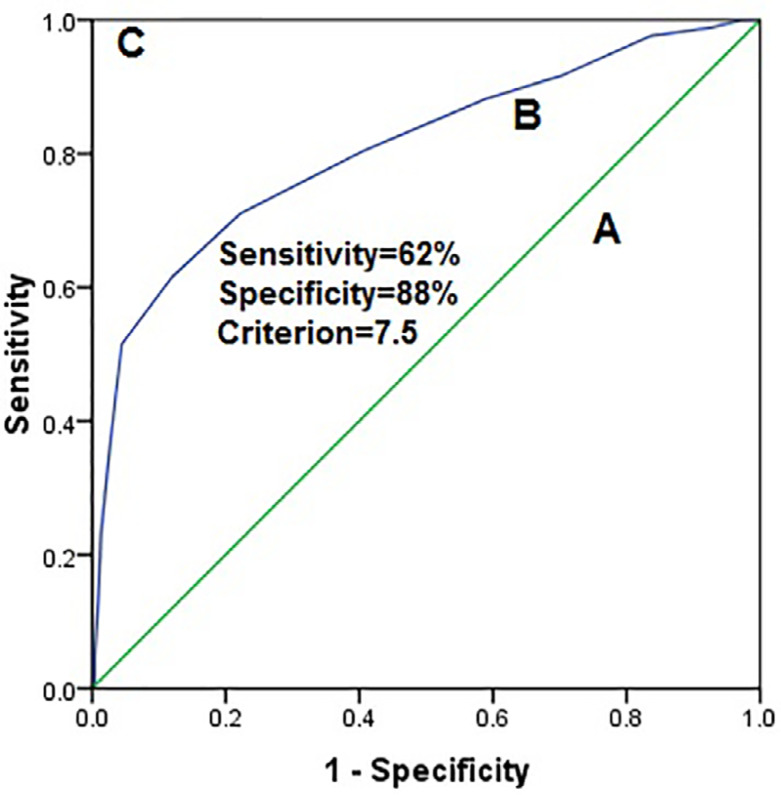
Receiver operator curves: A. No discrimination AUC = 0.5]; B. Experimental test (Mizan Sleep Quality and Sleep Hygiene Index total score) 0.81; p<.001]; C. Perfect test (AUC = 1) in Ethiopian university students.

The concurrent validity of the MiSQuaSHI as assessed by the ROC curve found an AUC of 0.81 (CI 0.76-0.85; *p*<.0001) (Figure 1). The sensitivity and specificity of the MiSQuaSHI at the cut-off score of 7.5 were 62% and 88%, respectively (Table 3), to screen poor sleep quality associated with poor sleep hygiene behavior.

**Table 3:** Sensitivity and specificity of the Mizan sleep quality and sleep hygiene index (MiSQuaSHI) for screening of poor sleep quality associated with poor sleep hygiene at each cut-off score in Ethiopian university students.

Cut-off score	Sensitivity	Specificity
0.5	1.00	0.03
1.5	0.99	0.08
2.5	0.98	0.16
3.5	0.92	0.30
4.5	0.88	0.41
5.5	0.80	0.59
6.5	0.71	0.78
7.5	0.62	0.88
8.5	0.51	0.96
9.5	0.36	0.97
10.5	0.23	0.99
11.5	0.12	0.99
12.5	0.05	1.00
13.5	0.01	1.00
15.0	0.00	1.00

### Divergent validity of the MiSQuaSHI

Significant correlations were found between GAD -7 and MiSQuaSHI total score (r=0.24), and MiSQuaSHI factor scores, i.e., sleep disturbances (r=0.13) and sleep hygiene (r=0.24). Similarly, significant correlations were found between PSS -10 and MiSQuaSHI total score (r=0.20) and MiSQuaSHI sleep hygiene (r=0.24).

### Internal consistency of the MiSQuaSHI

The Cronbach’s alpha was 0.70, while the Cronbach’s alpha (if item deleted) varied from 0.64-0.70. The internal homogeneity test of correlations between the item and the total score varied from 0.28-0.73 (*p*<0.01) (Table 2).

### Factor analysis of the MiSQuaSHI

**Figure 2:**
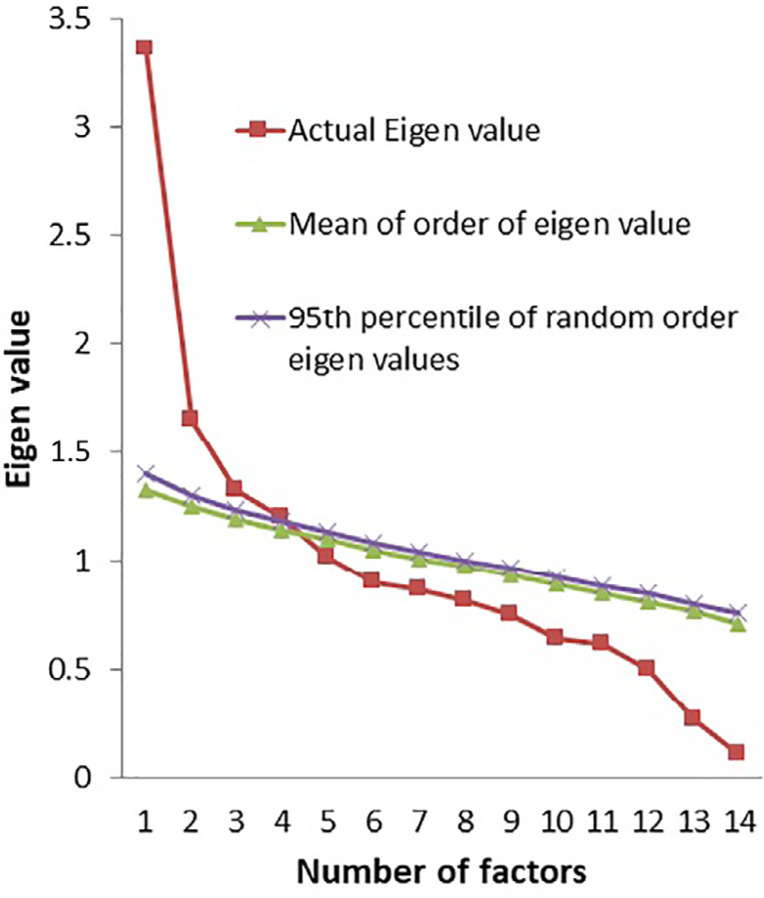
Parallel analysis sequence plot of the Mizan sleep quality and sleep hygiene index (MiSQuaSHI) in Ethiopian university students.

The sample size was adequate for factor analysis as indicated by diagonal elements of the anti-image matrix (0.52-0.92) and Kaiser-Meyer-Olkin test of sampling adequacy (KMO) (0.74)^23^. The condition of the absence of singularity in the MiSQuaSHI scores was indicated by Bartlett’s test of sphericity (<.001)^23^. The MiSQuaSHI scores did not have multi-collinearity as implied by the determinant score of 0.24, which was more than 0.00001^23^. The communality for MiSQuaSHI items (except for Item-10) were high, implying that the extracted factors explained a significant ratio of the item’s variance (Table 2)^23^. Most of the inter-item correlations between MiSQuaSHI scores were significant^23^. The results of the factor extraction measures were inconsisent. Kaiser’s criteria (Eigenvalue>1) and scree plot (factors above the point of inflection) found 5 factors (Table 4), cumulative variance rule (>40%) extracted 3 factors (Table 4) and parallel analysis (Monte Carlo PA) revealed a 4-factor model (Figure 2). CFA revealed that none of the tested models had an absolute fit to the data, i.e., non-significant χ^2^ test (Table 5). However, the 5-F model (Table 5) showed the best fit with the lowest values for RMSEA, RMR, χ^2^ and χ^2^/df, and highest values for IFI, CFI, and GFI (Table 5). The average loadings on the 1- factor, 2-factor, 3-factor, 4-factor, and 5-factor models were 0.32, 0.46, 0.43, 0.53, and 0.57, respectively.

**Table 4:** Summary of the factor extraction measures used in exploratory factor analysis of the Mizan Sleep Quality and Sleep Hygiene Index (MiSQuaSHI) in Ethiopian university students.

Number of Factors	Eigenvalue	Cumulative Variance Explained (%)	Above point of inflection on scree plot	Decision to extract		
				Kaiser's criteria (Eigenvalue≥1)	Cumulative variance rule (>40%)	Scree test
1	3.35	23.94	Yes	√	√	√
2	1.64	35.70	Yes	√	√	√
3	1.32	45.13	Yes	√	√	√
4	1.20	53.72	Yes	√	Χ	√
5	1.02	60.99	Yes	√	Χ	√
6	.90	67.40	No	Χ	Χ	Χ

√: indicates extraction criteria fulfilled; Χ: indicates otherwise.

**Table 5:** Fit statistics of the Mizan sleep quality and sleep hygiene index (MiSQuaSHI) in Ethiopian university students.

Models	PNFI	IFI	CFI	GFI	RMR	RMSEA	χ^2^	df	p	χ^2^/df
1-F	.68	.85	.85	.90	.02	.08 (.07-.09)	280.67	77	.00	3.64
2-F	.73	.92	.92	.94	.01	.06 (.05-.07)	193.04	76	.00	2.54
3-F	.70	.91	.90	.93	.01	.07(.06-.08)	206.23	74	.00	2.79
4-F	.72	.97	.97	.96	.01	.04 (.02-.05)	110.86	71	.01	1.56
5-F	.69	.98	.98	.97	.01	.03(.01-.04)	91.58	67	.02	1.37

PNFI: Parsimony normed fit index; IFI: Incremental fit index; CFI: Comparative fit index; GFI: Goodness of fit index; RMR: Root mean square residual; RMSEA: Root mean square error of approximation.

## DISCUSSION

The results support the validity of the newly developed MiSQuaSHI with satisfactory internal consistency, divergent validity, concurrent validity, item analysis, and structural validity in a sample of Ethiopian university students. The structural validity of the MiSQuaSHI in this non-clinical population of Ethiopian university students is favored by the absence of the ceiling and the floor effects. The variance accountability even at the lowest and highest scores of the MiSQuaSHI measurements suggests that it can be applied in the assessment of group differences and interventional studies^21^^,^^24^. This potential applicability is also supported by the absence of significant issues of skewness and kurtosis in the MiSQuaSHI total score^24^.

Furthermore, the results support the discriminative or the known-group validity of MiSQuaSHI. Sleep disturbances including insomnia are associated with anxiety^25^. The MiSQuaSHI total score, factor scores (i.e., sleep disturbances and sleep hygiene) and eight-item scores were higher in students with moderate-severe anxiety levels compared to those with no anxiety (normal) (Table 2). The fact that MiSQuaSHI total, as well as the factor scores, were significantly higher in students with moderate-severe anxiety than normal (no anxiety) students support the validity of the 2-factor model of the MiSQuaSHI as well as the composite construct of the tool.

Sleep is implicated to play an important role in almost all the physiological systems of the body^26^, and therefore, findings of a recent meta-analysis that found that there is a high prevalence of poor sleep quality among Ethiopians is a cause of concern for health and well-being^27^. Many sections of the Ethiopian population are at an increased risk of sleep problems such as university students, substance users, Khat-chewing pregnant women, people on anti-retroviral therapy for AIDS, etc.^27^^-^^30^. In such a circumstance, it is important to validate and establish sleep questionnaire tools among Ethiopians. Some of the previous studies have investigated psychometric validation of questionnaire tools such as the Pittsburgh sleep quality index, LSEQ-M, ESS, etc.^17^^,^^22^^,^^31^. However, this is the first study to report the development and validation of a new sleep questionnaire to assess both sleep quality as well as sleep hygiene in any demographics of the Ethiopian population.

LSEQ-M and its previous versions are cross-culturally validated tools to measure sleep disturbances including insomnia in clinics during pharmacological investigations as well as in non-clinical populations^17^^,^^32^^,^^33^. The AUC of the MiSQuaSHI with the concurrent measure of the LSEQ-M showed a moderate-high accuracy range^34^. It may not be out of context to propose that future studies should investigate the potential applicability of the MiSQuaSHI in different populations in both clinical and non-clinical settings. Poor sleep has been reported to be associated with stress and anxiety in Ethiopian university students^35^. Therefore, correlations of the MiSQuaSHI with the measures of the stress and anxiety (associated but distinct constructs) were estimated to assess its divergent validity^16^^,^^18^. The significant but weak level of correlations of the MiSQuaSHI scores with the stress and anxiety measures favor its divergent validity in this study among Ethiopian students.

MiSQuaSHI has adequate internal consistency as indicated by the Cronbach’s alpha test. The higher internal consistency of the MiSQuaSHI than that of the SHI^15^^,^^36^ further support the justification of the composite construct of MiSQuaSHI. The negligible variations in the Cronbach’s alpha value of the MiSQuaSHI on deleting items suggest that all items are relevant for its consistency. The internal homogeneity test of correlations between the item and the total scores of the MiSQuaSHI were significant for all items. Therefore, both consistency and homogeneity favor validation of the MiSQuaSHI in this population of Ethiopian university students.

The results of tests of the factor extraction in EFA were inconsistent with suggestions to retain 3-5 factors in the model. Two more models were assessed for fit in CFA. A 2-factor model was assessed based on theoretical considerations of the MiSQuaSHI being composed of items designed to measure sleep disturbances and sleep hygiene based on ICSD-R criteria^15^^,^^4^. Although model fit indices favored 5-factor over other models, the 2-factor model had an acceptable fit. Moreover, it had the highest value for PNFI, a parsimony-based model fit parameter. Previous studies support the use of parsimonious models in conditions of insignificant difference in model fit indices^37^^,^^38^. Therefore, the application of the 2-factor model is indicated. Moreover, future studies with multi-center data may help further establish the factorial validity of the MiSQuaSHI.

The limitations of the study include non-application of the gold-standard measure of the polysomnography for concurrent validity, a smaller number of female participants and sampling from only one university. Four sleep quality-assessing items of the MiSQuaSHI may not adequately address all aspects. Therefore, further research to explore expansion of items to comprehensively take appraisal of sleep quality across different socio-demographics may be needed. Future works to assess concurrent validation of the MiSQuaSHI employing polysomnography are needed. As evident from the participant characteristics, the study sample comprised of students with different linguistic ethnicities coming from different parts of Ethiopia. Future multi-centric studies with longitudinal designs to investigate measurement invariance of the 2-factor structure across socio-demographic characteristics and time is needed.

In spite of the abovementioned limitations, some of the merits of this study are worth mentioning. Evidence for adequate psychometric validity for the MiSQuaSHI was found. MiSQuaSHI is the first tool to assess sleep disturbances and sleep hygiene together in a single tool. Moreover, it is also the first sleep tool to be developed and validated in Ethiopian university students. Sleep problems are prevalent in Ethiopians university students, but the target population has limited access to sleep medicine professionals and sleep laboratories.

## CONCLUSION

Evidence for psychometric validation of the MiSQuaSHI was found in the Ethiopian university students. The MiSQuaSHI can be used to assess poor sleep quality associated with poor sleep hygiene practices in adolescents and young adults in general, as well as in the college/university attending population of these age groups. The MiSQuaSHI may help in targeted and easy screening of vulnerable groups of students during their university health center visits.

## SUPPLEMENT I. MIZAN SLEEP QUALITY AND SLEEP HYGIENE INDEX (MISQUASHI)

**Scoring guideline**

**1. Question numbers 1, 2, 5-13, 14 (a-d) are dichotomous and scored ‘0’ for ‘No’ and ‘1’ for ‘Yes’.**

**2. Question numbers 3 and 4 have a score range of 0-3; where a score of 0 is assigned for a response of ‘never’ and 3 is assigned for a response of ‘more than a year’/’more than four days’.**

**3. Scores for all the four sub-items (a-d) of question 14 are added. This sum score is converted into a single dichotomous score according to the following:**

i. 0-1 is coded as ‘0’

ii. 2-4 is coded as ‘1’

**4.** Finally, scores of all the dichotomously scored items (1, 2, 5-14) and structured categorical items (3, 4; these two items have a score range of 0-3; where a score of 0 is assigned for a response of ‘never’ and 3 is assigned for a response of ‘more than a year’/’more than four days’), are pooled to generate MiSQuaSHI total score in the range of 0-18. Higher scores indicate the possibility of poor sleep quality associated with poor sleep hygiene. A cut-off of 7.5 (if accompanied with a score, of ‘yes’ at least for question number-1 from the sleep disturbance section) was found to indicate presence of poor sleep quality associated with poor sleep hygiene in the present study on Ethiopian university students.
